# Control Gain Determination Method for Robust Time-Delay Control of Industrial Robot Manipulators Based on an Improved State Observer

**DOI:** 10.3390/s25185812

**Published:** 2025-09-17

**Authors:** Yu Chen, Jianwan Ding, Tianchang Xu, Yanbing Liu

**Affiliations:** School of Mechanical Science and Engineering, Huazhong University of Science and Technology, Wuhan 430074, China; d202180318@hust.edu.cn (Y.C.); d202380277@hust.edu.cn (T.X.); d202180319@hust.edu.cn (Y.L.)

**Keywords:** robotic manipulator control, robust time-delay control, control gain optimization, state observer

## Abstract

High-precision control of robotic manipulators plays a vital role in improving the efficiency and quality of industrial manufacturing. However, the inherent nonlinear and time-varying characteristics of robotic systems make high-accuracy control a challenging task, and external noise interference further complicates reliable state estimation. Conventional time-delay control methods often involve computationally intensive procedures for gain determination and are limited in their ability to suppress noise effectively. To overcome these limitations, this paper proposes a robust time-delay control strategy based on an improved state observer. By deriving a linearized form of the dynamic model, an offline computation scheme for control gain determination is developed, which eliminates the need for additional tuning parameters and simplifies the design process. Furthermore, the proposed state observer integrates model reference estimation with noise suppression techniques, enabling accurate acquisition of joint states and improving system robustness under noisy conditions. Experimental results validate the effectiveness of the proposed method, showing that it can efficiently determine control gains and significantly outperform existing advanced approaches in terms of trajectory tracking accuracy and overall control performance.

## 1. Introduction

Robotic manipulators, with their high precision, efficiency, and reliability, have become core equipment driving the automation and intelligent transformation of industrial manufacturing [1]. However, their dynamics are characterized by nonlinearities, strong couplings, and time-varying complexities, while unmodeled uncertainties such as joint friction, load variations, and external disturbances are also present. These factors not only increase the difficulty of dynamic modeling and parameter identification but also pose severe challenges to control accuracy and system stability [2].

In practical applications, control challenges mainly arise from two aspects: the complex dynamic model and multi-joint coupling effects impose a large computational burden for real-time implementation, while uncertainties and higher-order states that are difficult to measure directly significantly affect control performance. Relying on additional sensors to measure these states introduces extra costs and reduces system reliability. Although various strategies such as sliding mode control [3,4,5], adaptive control [6], fuzzy logic control [7], and neural network control [8,9,10,11] have been proposed, these methods often depend heavily on accurate modeling or incur excessive computational costs, limiting their widespread application in industrial practice.

Time-Delay Control (TDC) is a simple yet effective approach that can compensate for dynamic uncertainties without relying on precise dynamic models, thereby avoiding complex computations. Owing to this advantage, TDC has been widely applied in robotic manipulators [12,13,14,15], motor drives [16,17], unmanned aerial vehicles [18], flexible robots [19,20,21], and general Lagrangian systems [22], demonstrating excellent control performance. The core idea of TDC is to estimate the system’s dynamic characteristics using delayed torque and acceleration information. This approach requires a sufficiently short sampling period and accurate measurements of joint accelerations. Nevertheless, existing TDC schemes still face two key challenges: determining the appropriate control gain $\bar{M}$ and obtaining accurate joint acceleration information without relying on additional sensors.

Previous studies have demonstrated that the more accurately the control gain $\bar{M}$ is determined, the higher the control precision and the faster the dynamic response of the system [23,24,25]. However, most existing TDC schemes still rely on experimental tuning to determine $\bar{M}$, typically adjusting it iteratively according to the desired error and using the smallest possible sampling period, as in the method proposed by Chang [26]. This approach heavily depends on engineering experience and requires considerable time for tuning. To improve automation, various adaptive adjustment algorithms have been proposed: Seung-Jae Cho [27] developed an automatic gain adjustment method based on an improved Nussbaum function; M. Jin [28] proposed a dynamic self-tuning algorithm based on sliding-mode error; and J. Baek [29] designed a novel adaptive sliding-mode control strategy. Although these methods partially enhance the automation of gain tuning, they still exhibit notable limitations, including sensitivity to noise, a large number of control parameters that increase design complexity, and adaptive laws that cannot respond promptly or accurately to tracking errors.

High-quality feedback signals are essential for high-performance motion control, but they are often corrupted by pulse-sampling noise, which degrades control quality. Most researchers estimate acceleration using numerical differentiation; for example, Wang et al. [30] calculated the acceleration $\ddot{q}$ from position signals *q* as ${\ddot{q}}_{\left(t-L\right)}=(q_{\left(t\right)}-2q_{\left(t-L\right)}+q_{\left(t-2L\right)})t_{s}^{-2}$. However, such numerical methods typically amplify noise significantly. To mitigate this, some studies have incorporated observers within the TDC framework. Chang et al. [31] proposed a model reference observer that replaces the complex nonlinear dynamics with a linear reference model, while Lee et al. [32] developed a time-delay observer using delayed terms to estimate system uncertainties. Nevertheless, these approaches still cannot fully suppress noise interference.

In recent years, intelligent computing methods have been increasingly introduced into the state prediction and control of complex dynamical systems. Cross-disciplinary approaches such as neural networks, deep learning, cognitive computing, and model predictive control (MPC) can automatically learn system characteristics from data, capture nonlinear relationships, and achieve high-precision prediction under unknown disturbances or complex noise environments. Ciria et al. [33] pointed out that the predictive processing framework unifies perception, cognition, and action into a single inference process, thereby enhancing the adaptability of cognitive robots. Chen et al. [34] demonstrated that the output recurrent LSTM (OR-LSTM) possesses long-term state prediction capability for nonlinear dynamical systems, remaining robust even under partial observation or complex oscillations. Peng et al. [35] applied cognitive computing and machine learning to predict flow status in flexible fluidic devices, achieving high accuracy with low computational cost. Hilal et al. [36] emphasized that cognitive dynamic systems (CDSs), through the perception–action cycle and intelligent integration, enable adaptive optimization of engineering systems. Song et al. [37] showed that MPC provides systematic optimization of control inputs and robustness guarantees complex, time-varying, and uncertain systems.

Inspired by the above cross-disciplinary studies, this paper proposes a robust time-delay control framework to enhance the performance of robotic manipulators under complex conditions such as multi-joint coupling, non-Gaussian noise, and load variations. To address the challenge of determining control gains, an optimization method based on a linearized dynamic model is introduced, which allows the control gains to be computed offline without additional tuning parameters, thereby avoiding the time-consuming and cumbersome calibration process by engineers. To tackle the difficulty of acquiring joint state information, a signal enhancement approach combining a model reference observer with a Kalman filter is further proposed, which significantly improves the robustness of the control system under noisy conditions. This method not only integrates the strengths of intelligent optimization and observer techniques but also enables high-precision and robust manipulator control in complex operating environments.

The structure of this paper is organized as follows: Section 2 presents the fundamental principles of time-delay control; Section 3 introduces the method for determining the control gains; Section 4 designs an improved state observer integrated with time-delay control; Section 5 describes the trajectory tracking experiments of the robotic manipulator and their results; Section 6 discusses the experimental findings; and Section 7 concludes the paper and summarizes the main conclusions.

## 2. Theory and Framework of Time-Delay Control

An n-DOF robotic manipulator is the most commonly used type in industrial applications, and its dynamics can be expressed as follows (1):

(1) $$\tau =M\left(q\right)\ddot{q}+V\left(q,\;\dot{q}\right)\dot{q}+G\left(q\right)+F\left(q,\;\dot{q}\right).$$

Here, τ denotes the joint torque, $M\left(q\right)$ is the inertia matrix, $V\left(q,\;\dot{q}\right)$ is the Coriolis matrix, $G\left(q\right)$ is the gravity matrix, and $F\left(q,\;\dot{q}\right)$ is the friction torque matrix. The variables $q,\;\dot{q},\;\ddot{q}$ represent the joint positions, velocities, and accelerations of the manipulator, respectively. Due to parameter perturbations and uncertainties, accurately obtaining the above dynamic matrices is challenging. To address this, Hsia et al. [38] proposed the Hsia formulation, as shown in (2), to account for the effects of uncertainties.

(2) $$\tau =\bar{M}\ddot{q}+N\left(q,\;\dot{q},\;\ddot{q}\right),$$

where

(3) $$N\left(q,\;\dot{q},\;\ddot{q}\right)≜\left[M\left(q\right)-\bar{M}\right]\ddot{q}+V\left(q,\;\dot{q}\right)\dot{q}+G\left(q\right)+F\left(q,\;\dot{q}\right).$$

Here, $N\left(q,\;\dot{q},\;\ddot{q}\right)$ represents the time-delay estimation (TDE), which accounts for all uncertainties and unmodeled dynamics. $\bar{M}$ is the estimated inertia matrix of $M\left(q\right)$ and also serves as the control gain matrix.

Based on the concept of TDC, $N\left(q,\;\dot{q},\;\ddot{q}\right)$ can be approximated. When the sampling time *L* is sufficiently small, the value of $N\left(q,\;\dot{q},\;\ddot{q}\right)$ at time *t* can be considered equal to its value at time *t* − *L*, that is:

(4) $${N\left(q,\;\dot{q},\;\ddot{q}\right)}_{\left(t\right)}≅{N\left(q,\;\dot{q},\;\ddot{q}\right)}_{\left(t-L\right),\;}$$

where _(*t*)_ and _(*t*−*L*)_ denote the values at time *t* and *t* − *L*, respectively.

From (2), it follows that:

(5) $${N\left(q,\;\dot{q},\;\ddot{q}\right)}_{\left(t-L\right)}=\tau_{\left(t-L\right)}-\bar{M}{\ddot{q}}_{\left(t-L\right).}$$

From (5), it can be seen that the TDE ${N\left(q,\;\dot{q},\;\ddot{q}\right)}_{\left(t\right)}$ at time *t* can be determined using only the torque value $\tau_{\left(t-L\right)}$ and the acceleration information ${\ddot{q}}_{\left(t-L\right)}$ at time *t* − *L*, thereby avoiding complex dynamic computations. The smaller the sampling time *L*, the higher the estimation accuracy of $N\left(q,\;\dot{q},\;\ddot{q}\right)$; however, an excessively small *L* increases the system’s sensitivity to noise. In industrial applications, *L* is typically set equal to the system’s sampling period *T* [39].

The position tracking error is defined as: $e_{\left(t\right)}=\;q_{d(t)}-q_{\left(t\right)}$, where $q_{d}$ is the desired joint trajectory. The time-delay control scheme can then be expressed as (6):

(6) $$\tau_{\left(t\right)}=\underset{Injection\;term}{\underbrace{\bar{M}\left({\ddot{q}}_{d\left(t\right)}+K_{v}{\dot{e}}_{\left(t\right)}+K_{p}e_{\left(t\right)}\right)}}+\underset{Time\;delay\;Estimation}{\underbrace{\tau_{\left(t-L\right)}-\bar{M}{\ddot{q}}_{\left(t-L\right)}}}.$$

Let: $u_{\left(t\right)}={\ddot{q}}_{d(t)}+K_{v}{\dot{e}}_{\left(t\right)}+K_{p}e_{\left(t\right)}$, which is used to inject the desired error dynamics into the closed-loop system, where $K_{v}=diag\left(K_{v1},\;K_{v2},\;\cdots ,\;K_{vn}\right)$ and $K_{p}=diag(K_{p1},\;K_{p2},\;\cdots ,\;K_{pn})$ are positive-definite velocity and position gain matrices, respectively.

By substituting (6) and (5) into (2), the error dynamics of the manipulator system can be written as:

(7) $${\ddot{e}}_{\left(t\right)}+K_{v}{\dot{e}}_{\left(t\right)}+K_{p}e_{\left(t\right)}=\varepsilon_{\left(t\right)},$$

where $\varepsilon_{\left(t\right)}=-{\bar{M}}^{-1}({N\left(q,\dot{q},\ddot{q}\right)}_{\left(t-L\right)}-{N\left(q,\dot{q},\ddot{q}\right)}_{\left(t\right)})$, which can be approximated as zero when the sampling time *L* is sufficiently small. According to the Lyapunov stability criterion, it is sufficient to prove that $\varepsilon_{\left(t\right)}$ is asymptotically bounded in order to conclude that the tracking error $e_{\left(t\right)}$ is also asymptotically bounded, thereby ensuring the stability of the manipulator system. The asymptotic boundedness of $\varepsilon_{\left(t\right)}$ requires not only that $\bar{M}$ is invertible but also that condition (8) is satisfied [27]:

(8) $$\left\|I-M^{-1}\bar{M}\right\|<1.$$

**Figure 1:**
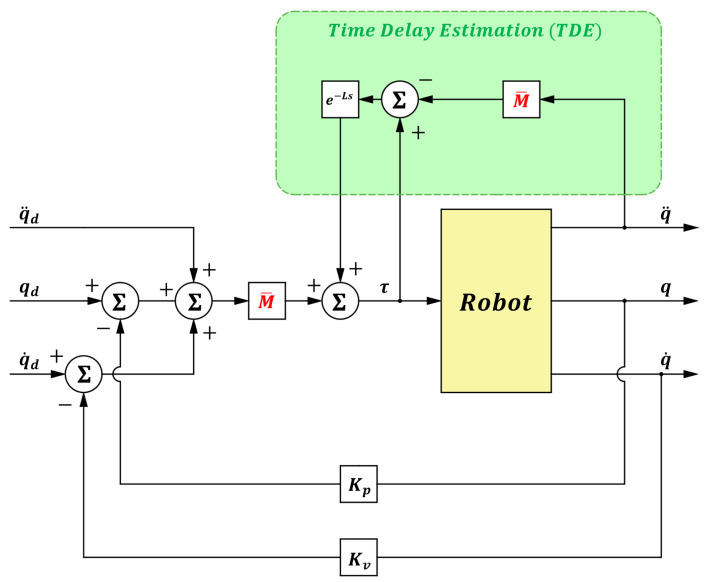
Block diagram of time-delay control.

The block diagram of the manipulator system under the time-delay control method is shown in Figure 1.

## 3. Design Method for Control Gains in Time-Delay Control

### 3.1. Linearized Control Gain Matrix Derivation

The dynamics of robotic manipulators can be described using the Lagrangian equation [40,41], where the Lagrangian function $L\left(q,\dot{q}\right)$ is defined as the difference between the system’s total kinetic energy $K\left(q,\dot{q}\right)$ and total potential energy P(q), that is, $L\left(q,\dot{q}\right)=K\left(q,\dot{q}\right)-P(q)$.

The dynamic equation can then be expressed by the Lagrangian formulation as (9):

(9) $$\tau =\frac{d}{dt}\frac{\partial L\left(q,\dot{q}\right)}{\partial \dot{q}}-\frac{\partial L\left(q,\dot{q}\right)}{\partial q}=\frac{d}{dt}\frac{\partial K\left(q,\dot{q}\right)}{\partial \dot{q}}-\frac{\partial K\left(q,\dot{q}\right)}{\partial q}+\frac{\partial P\left(q\right)}{\partial q}.$$

Since $\frac{\partial K\left(q,\dot{q}\right)}{\partial \dot{q}}$ is a function of both *q* and $\dot{q}$, its derivative with respect to time *t* can be decomposed into two parts, namely:

(10) $$\frac{d}{dt}\frac{\partial K\left(q,\dot{q}\right)}{\partial \dot{q}}=\frac{\partial }{\partial \dot{q}}\frac{\partial K\left(q,\dot{q}\right)}{\partial \dot{q}}\ddot{q}+\frac{\partial }{\partial q}\frac{\partial K\left(q,\dot{q}\right)}{\partial \dot{q}}\dot{q}.$$

Substituting (10) into (9) yields:

(11) $$\tau =\underset{\bar{M}}{\underbrace{\frac{\partial }{\partial \dot{q}}\frac{\partial K\left(q,\dot{q}\right)}{\partial \dot{q}}}}\ddot{q}+\underset{N\left(q,\dot{q},\ddot{q}\right)}{\underbrace{\frac{\partial }{\partial q}\frac{\partial K\left(q,\dot{q}\right)}{\partial \dot{q}}\dot{q}-\frac{\partial K\left(q,\dot{q}\right)}{\partial q}+\frac{\partial P\left(q\right)}{\partial q}}}.$$

By comparing (2) and (11), it can be seen that with the Lagrangian formulation, the control gain matrix $\bar{M}$ can be extracted in a linearized form. To facilitate the identification of the control gain, (11) can be reformulated as:

(12) $$\begin{array}{c} \tau =\underset{\bar{M}}{\underbrace{\frac{\partial }{\partial \dot{q}}\frac{\partial K\left(q,\dot{q}\right)}{\partial \dot{q}}}}\ddot{q}+\underset{C}{\underbrace{\frac{\partial }{\partial q}\frac{\partial K\left(q,\dot{q}\right)}{\partial \dot{q}}}}\dot{q}\underset{Gq+X}{\underbrace{-\frac{\partial K\left(q,\dot{q}\right)}{\partial q}+\frac{\partial P\left(q\right)}{\partial q}}}\\ =\bar{M}\ddot{q}+C\dot{q}+Gq+X \end{array}.$$

where $\bar{M}$ denotes the control gain matrix, while *C*, *G*, and *X* are auxiliary variables used to identify $\bar{M}$.

### 3.2. Identification Method of the Control Gain Matrix

In this paper, the proposed identification method is applicable to both single-joint and multi-joint control systems, and the entire identification process can be performed offline. Before performing the identification, the following preliminary work is required:

Given the task trajectories $q_{d}\left(s\right),\;{\dot{q}}_{d}\left(s\right),\;{\ddot{q}}_{d}\left(s\right),\;(s=0,\cdots ,k)$, the corresponding joint torques $\tau_{i}(s)$ and the inertia matrix $M\left(q_{d}(s)\right)$ can be computed using the Newton–Euler method [42]. Here, *s* denotes the discrete nodes, and the torque values $\tau_{i}(s)$ can be approximately considered equal to those obtained from the actual dynamics in (1).

To obtain the most accurate control gain matrix $\bar{M}$, Equation (12) should be made to match the actual robot dynamics (Equation (1)) as closely as possible; that is, the torque values calculated from Equation (12) should approximate the $\tau_{i}(s)$ computed during the preliminary work.

**Case 1:** For a single-joint control system, the control gain $\bar{M}=diag({\bar{M}}_{1},\;{\bar{M}}_{2},\cdots ,\;{\bar{M}}_{n})$, and the auxiliary variables *C*, *G*, and *X* are also diagonal matrices. In this case, Hsia et al. [43] further expressed the system stability constraint in (8) as (14), where $\rho_{i}$ denotes the lower bound of the eigenvalues of $M\left(q_{d}(s)\right)$.

(13) $${\bar{M}}_{i}\leq 2\rho_{i}$$

**Figure 2:**
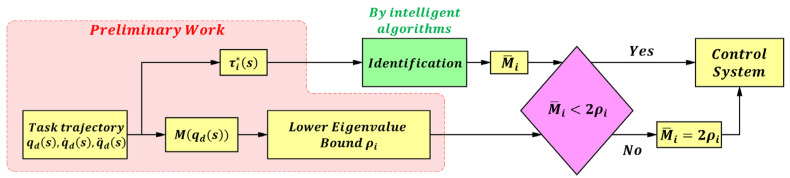
Identification flowchart of a single-joint control system.

The identification procedure for a single-joint control system is shown in Figure 2.

In this case, a sequential identification scheme is adopted to determine each control gain ${\bar{M}}_{i}$, that is, the identification is performed successively from joint 1 to joint *n*. From Equation (12), the control gain ${\bar{M}}_{i}$ of each joint, along with the auxiliary variables $C_{i},G_{i},X_{i}$, determines the torque value $\tau_{i}^{*}$. Taking the *i*-th joint as an example (i=1,⋯,n):

(14) $$\tau_{i}^{*}\left(s\right)={\bar{M}}_{i}{\ddot{q}}_{di}\left(s\right)+C_{i}{\dot{q}}_{di}\left(s\right)+G_{i}q_{di}\left(s\right)+X_{i}.$$

**Figure 3:**
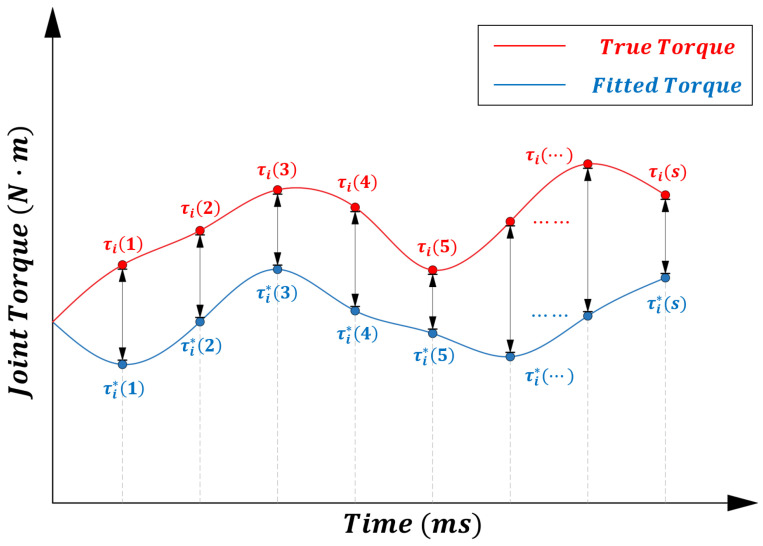
Optimization objective of the constrained model for a single-joint control system.

From the above equation, it can be seen that by adjusting the control gain ${\bar{M}}_{i}$ and the auxiliary variables, the fitted torque $\tau_{i}^{*}(s)$ can be made as close as possible to the actual dynamic torque $\tau_{i}(s)$. This gain adjustment process can be transformed into an optimization problem, where the objective function is given by Equation (15), and the design variables are ${\bar{M}}_{i},\;C_{i},\;G_{i},\;X_{i}$, see Figure 3.

(15) $$\min J=\sum_{s=1}^{k}\frac{\left|\tau_{i}\left(s\right)-\tau_{i}^{*}\left(s\right)\right|}{k}$$

**Case 2:** For a multi-joint control system, the coupling effects between joints must be considered. In this case, the control gain is given by $\bar{M}=\left[\begin{array}{ccc} {\bar{M}}_{11} & \ldots & {\bar{M}}_{1n}\\ \vdots & \ddots & \vdots \\ {\bar{M}}_{n1} & \ldots & {\bar{M}}_{nn} \end{array}\right]$, and the auxiliary variables *C*, *G*, and *X* have the same structure.

**Figure 4:**
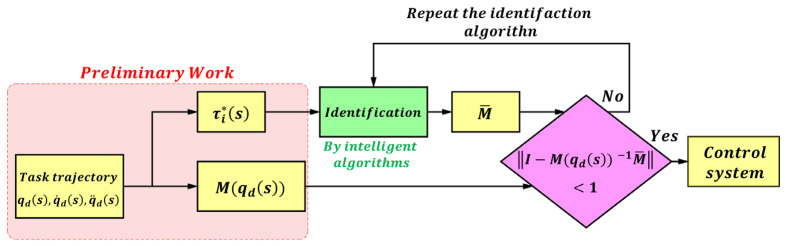
Identification flowchart of the multi-joint control system.

The identification process for the multi-joint control system is shown in Figure 4.

In this case, a global identification scheme is adopted to determine the control gain matrix $\bar{M}$. From Equation (12), we obtain:

(16) $$\tau^{*}\left(s\right)=\bar{M}{\ddot{q}}_{d}\left(s\right)+C{\dot{q}}_{d}\left(s\right)+Gq_{d}\left(s\right)+X,$$

where $\tau^{*}\left(s\right)=[\tau_{1}^{*}\left(s\right),\cdots ,\tau_{n}^{*}\left(s\right)]$. The decision variables for this optimization problem are $\bar{M}$, *C*, *G* and *X*. The optimization objective is given by (17), where $w_{i}$ denotes the weight coefficient of the *i*-th joint. If higher accuracy is required for a specific joint, its weight coefficient can be appropriately increased.

(17) $$\min J=\sum_{i=1}^{n}\left(w_{i}\times \frac{1}{k}\sum_{s=1}^{k}\left|\tau_{i}\left(s\right)-\tau_{i}^{*}\left(s\right)\right|\right).$$

The above optimization problem can be solved using various intelligent algorithms. For illustration purposes, this paper takes a multi-joint system as an example and selects the Particle Swarm Optimization (PSO) algorithm as the demonstration method. The basic idea is to treat the control gain $\bar{M}$ and auxiliary variables *C*, *G*, and *X* as particles within the swarm, where each particle *i* is represented by a position vector $x_{i}$ and a velocity vector $v_{i}$. The fitness function *J* is defined in (15) or (17). During the search process, each particle continuously updates its state by tracking its own historical best position $p_{i}$ and the global best position of the swarm $p_{g}$. The iterative update rules for the particle’s velocity and position are as follows:

(18) $$v_{i}^{\left(k+1\right)}=w_{PSO}\cdot v_{i}^{\left(k\right)}+c_{1}\cdot r_{1}\cdot \left(p_{i}-x_{i}^{k}\right)+c_{2}\cdot r_{2}\cdot \left(p_{g}-x_{i}^{k}\right),$$

(19) $$x_{i}^{\left(k+1\right)}=x_{i}^{k}+v_{i}^{\left(k+1\right)},$$

where $w_{PSO}$ is the inertia weight of the particle swarm algorithm, $c_{1}$ and $c_{2}$ are acceleration factors, and $r_{1},r_{2}\in [0,1]$ are random numbers. The term $\left(p_{i}-x_{i}^{k}\right)$ represents the particle’s update along its personal best direction, while $\left(p_{g}-x_{i}^{k}\right)$ represents the update along the global best direction.

**Figure 5:**
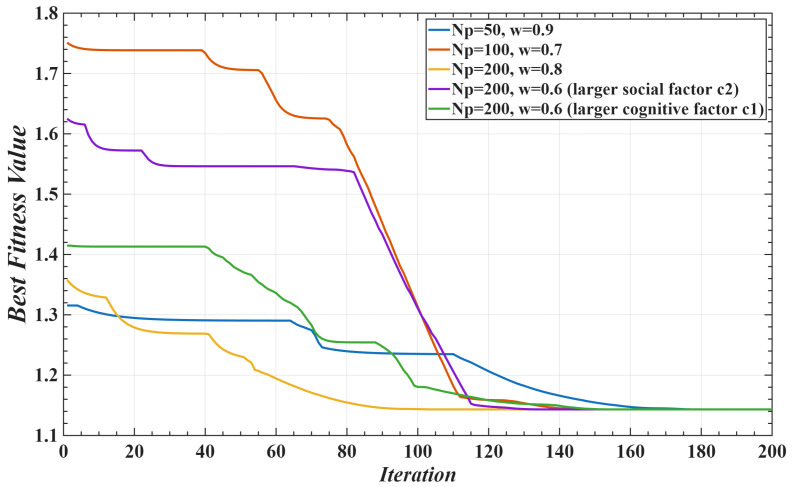
PSO convergence curves under different initial conditions.

In the implementation, the PSO parameters were set as follows: population size of 30, maximum number of iterations of 200, inertia weight $w_{PSO}=0.7$, and acceleration factors $c_{1}=c_{2}=1.5$. The convergence criterion was defined such that the algorithm is considered converged if the change in the fitness function remains smaller than 10^−5^ for 20 consecutive iterations. To evaluate the reliability of convergence, 10 independent runs were performed under different random initial conditions. The results showed that the variance of the final fitness values was below 2%, indicating good stability and convergence of the algorithm. The average computation time was approximately 0.2 s, which is sufficient to meet offline optimization requirements. In addition, the differences in experimental results under varying initialization ranges were found to be minor, further demonstrating the insensitivity of the method to initial conditions, see Figure 5.

### 3.3. Simulation Validation of Gain Identification

**Figure 6:**
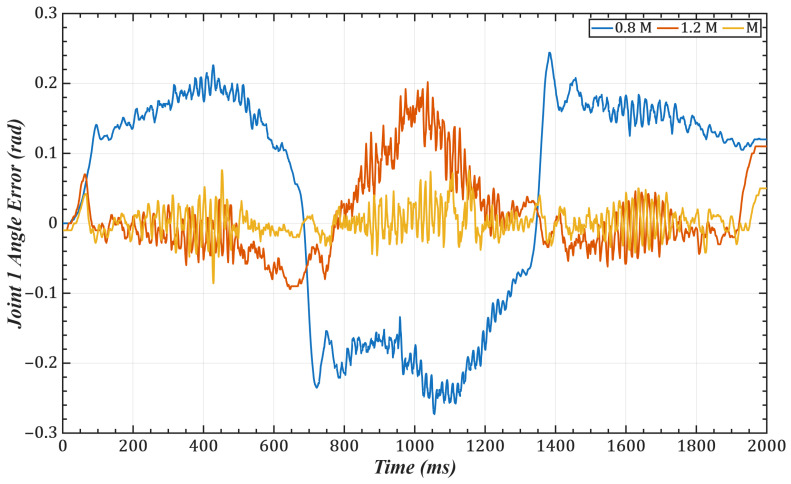
Tracking error of Joint 1.

**Figure 7:**
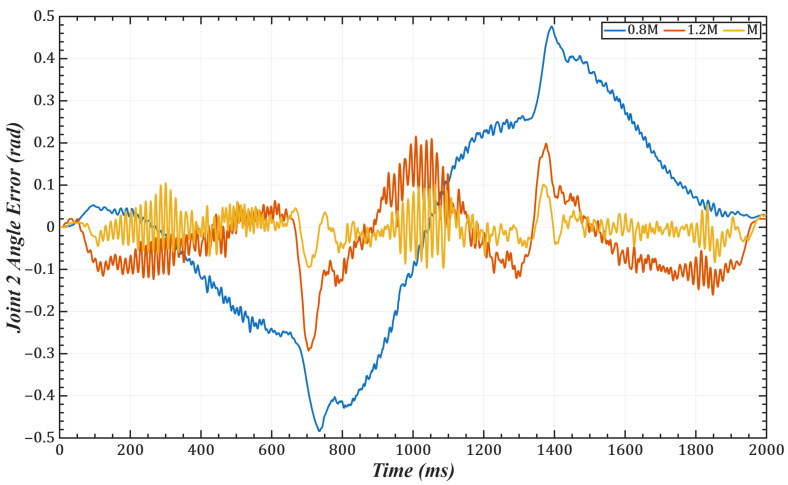
Tracking error of Joint 2.

To validate the effectiveness of the identification scheme proposed in our paper, we conducted trajectory tracking simulations on joints 1 and 2 of a 6-DOF robotic manipulator. The task trajectory was set as: $q_{d(t)}=\frac{\pi }{6}\sin\left(\frac{\pi }{4}t\right)-\frac{\pi }{12}\sin\left(\frac{\pi }{6}t\right)$. with control gains selected as ${0.8\bar{M}}_{i},\;{1.2\bar{M}}_{i},\;{\bar{M}}_{i}(i\;=\;1,2)$. The joint errors are shown in Figure 6 and Figure 7, and the corresponding mean and root-mean-square (RMS) errors are listed in Table 1 and Table 2. The results indicate that the identified control gains can accurately match the manipulator’s dynamic characteristics, thereby significantly improving trajectory tracking accuracy.

## 4. Improved State Observer Design

In industrial systems, typically only the joint position signals *q* can be obtained. To acquire the system state information *x* $\left(x={\left[\begin{array}{cc} q & \dot{q} \end{array}\right]}^{T}\right)$, this paper constructs a linear reference model to replace the real dynamics: $\dot{z}=A_{m}z+B_{m}r$, where *z* is the observed state ($z={\left[\begin{array}{cc} z_{1} & z_{2} \end{array}\right]}^{T}$). The observer error is defined as $e_{m}=z-x$. To achieve $e_{m}\equiv 0$, it is required that ${\dot{e}}_{m}=A_{m}e_{m}$. Accordingly, Equation (7) can be rewritten as Equation (20), where $A_{m}=\left[\begin{array}{cc} 0 & I\\ -K_{p} & -K_{v} \end{array}\right]$.

(20) $${\dot{x}}_{d}-\dot{x}=\underset{A_{m}}{\underbrace{\left[\begin{array}{cc} 0 & I\\ -K_{p} & -K_{v} \end{array}\right]}}\left(x_{d}-x\right)$$

By combining Equation (20) with ${\dot{e}}_{m}=A_{m}e_{m}$, Equation (21) can be obtained, where $B_{m}r={\dot{x}}_{d}-A_{m}x_{d}$:

(21) $$\dot{z}=A_{m}z+\underset{B_{m}r}{\underbrace{\left({\dot{x}}_{d}-A_{m}x_{d}\right)}}.$$

Equation (21) represents the linear reference model. The model reference observer is constructed as in Equation (22), where $C=[\begin{array}{cc} I & 0 \end{array}]$ and $F={\left[\begin{array}{cc} F_{1} & F_{2} \end{array}\right]}^{T}$:

(22) $$\dot{z}=A_{m}z+B_{m}r+C\left(z-x\right).$$

From Equation (22), the specific structure of the model reference observer can be further derived as follows:

(23) $$\left\{\begin{array}{c} {\dot{z}}_{1}=z_{2}+F_{1}\left(z_{1}-q\right)\\ {\dot{z}}_{2}={\ddot{q}}_{d}-K_{v}\left(z_{2}-{\dot{q}}_{d}\right)-K_{p}\left(z_{1}-q_{d}\right)+F_{2}\left(z_{1}-q\right) \end{array}\right..$$

**Figure 8:**
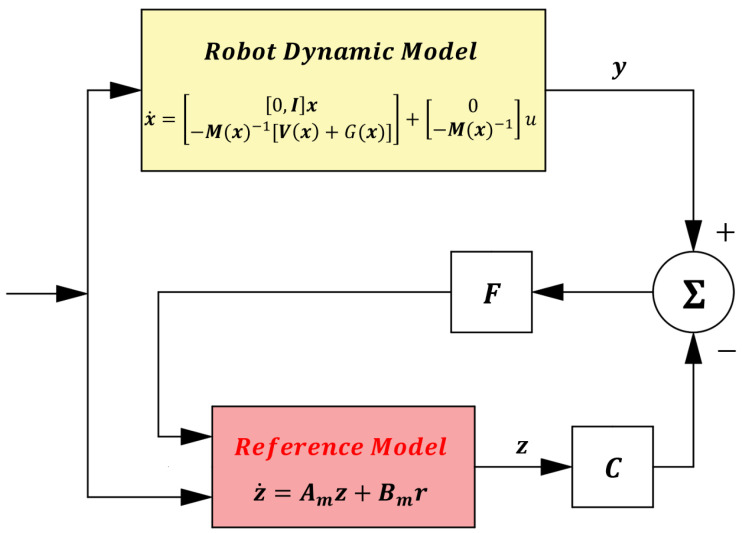
Schematic of the Model Reference Observer.

The structure of this observer is shown in Figure 8, which can effectively estimate the state information of the robotic manipulator.

**Figure 9:**
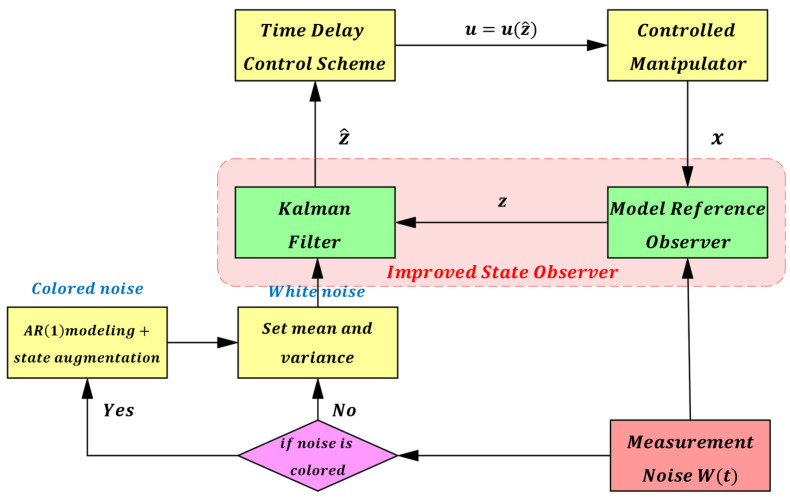
Schematic of the Improved State Observer.

To overcome the interference from measurement noise *W(t)*, a Kalman filter is introduced to improve the model reference observer in this paper. The improved state observer within the overall control scheme is illustrated in Figure 9. The state-space equation of the model reference observer can be expressed as follows (24):

(24) $$\left[\begin{array}{c} {\dot{z}}_{1}\\ {\dot{z}}_{2} \end{array}\right]=\left[\begin{array}{cc} 0 & 1\\ 0 & 0 \end{array}\right]\left[\begin{array}{c} z_{1}\\ z_{2} \end{array}\right]+\left[\begin{array}{c} 0\\ 1 \end{array}\right]{\dot{z}}_{2}.$$

The Euler method can be used to discretize Equation (24), i.e., $\dot{z}\left(kT\right)=\frac{z\left[\left(k+1\right)T\right]-z(kT)}{T}$. The corresponding state-space equation is then expressed as follows (25):

(25) $$\left[\begin{array}{c} z_{1}\left[\left(k+1\right)T\right]\\ z_{2}\left[\left(k+1\right)T\right] \end{array}\right]=\left[\begin{array}{cc} 1 & T\\ 0 & 1 \end{array}\right]\left[\begin{array}{c} z_{1}\left(kT\right)\\ z_{2}\left(kT\right) \end{array}\right]+\left[\begin{array}{c} 0\\ T \end{array}\right]{\dot{z}}_{2}\left(kT\right).$$

Measurement noise *W(t)* can generally be classified into two categories: Gaussian white noise and colored noise. To analyze its characteristics, this study approximates the output of the manipulator’s joint angle sensors under no-load conditions as primarily reflecting measurement noise, from which raw data are obtained for modeling. When the noise can be approximated as white noise, its mean and variance are calculated, and the statistical properties of the measurement noise *W(t)* are set accordingly. If the noise exhibits colored characteristics, it is modeled as an *AR*(1) process, as expressed in (26). In addition, if the noise contains excessively high-frequency components, a low-pass filter can be applied to further suppress the undesired disturbances.

(26) $$W\left(k+1\right)=\alpha W\left(k\right)+\eta \left(k\right),$$

where α controls the correlation of the colored noise (0<α<1), and $\eta \left(k\right)$ is zero-mean white noise with variance $\sigma^{2}$. The parameters are obtained by fitting the no-load sensor data. In practical applications, if the noise has a nonzero mean, it can be estimated or removed prior to filtering, thereby ensuring the validity of the zero-mean assumption for $\eta \left(k\right)$.

In the filter design, the colored noise is modeled as an additional state and incorporated into the system state vector, resulting in the augmented state: $z_{aug}(k)=\left[z(k);W(k)\right]$. The corresponding discrete state-space equation is as follows:

(27) $$z_{aug}\left(k+1\right)=\left[\begin{array}{ccc} 1 & T & 0\\ 0 & 1 & 0\\ 0 & 0 & \alpha \end{array}\right]{\;z}_{aug}\left(k\right)+\left[\begin{array}{c} 0\\ T\\ 1 \end{array}\right]\;\eta \left(k\right).$$

**Figure 10:**
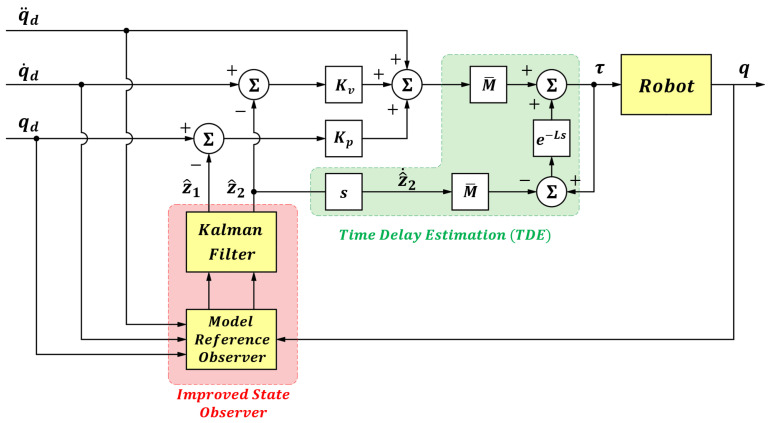
Overall Control System Block Diagram.

After obtaining the statistical model of the measurement noise, it is incorporated into the Kalman filter for state estimation. Through filtering, the augmented state $\hat{z}=\left[\begin{array}{c} {\hat{z}}_{1}\\ {\hat{z}}_{2} \end{array}\right]$ can be regarded as the true system state $x=\left[\begin{array}{c} q\\ \dot{q} \end{array}\right]$. Based on this, the time-delay control law (6) is reformulated as (28), and the overall block diagram of the improved control system is shown in Figure 10.

(28) $$\tau_{\left(t\right)}=\bar{M}\left({\ddot{q}}_{d\left(t\right)}+K_{v}\left({\dot{q}}_{d\left(t\right)}-{\hat{z}}_{2\left(t\right)}\right)+K_{p}\left(q_{d\left(t\right)}-{\hat{z}}_{1}\right)\right)+\tau_{\left(t-L\right)}-\bar{M}\;{\dot{\hat{z}}}_{2\left(t-L\right)}$$

**Figure 11:**
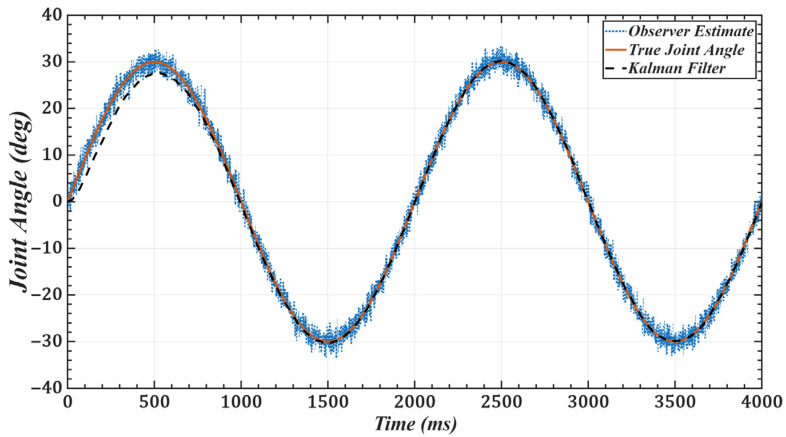
Joint 1 position with Gaussian white noise: desired, noisy observer, and filtered.

**Figure 12:**
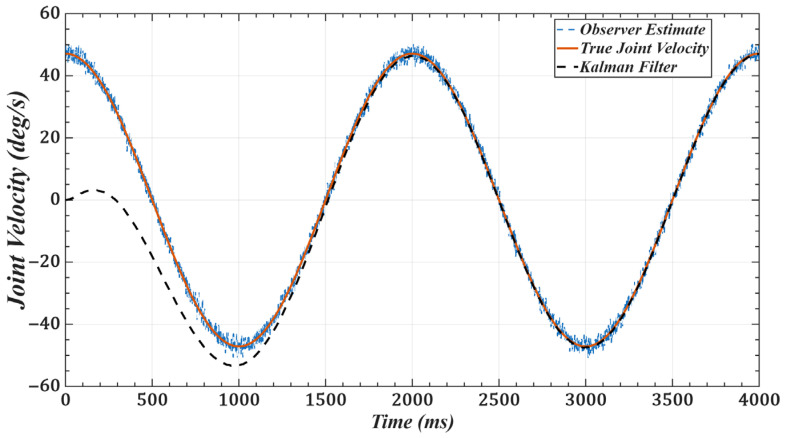
Joint 1 velocity with Gaussian white noise: desired, noisy observer, and filtered.

**Figure 13:**
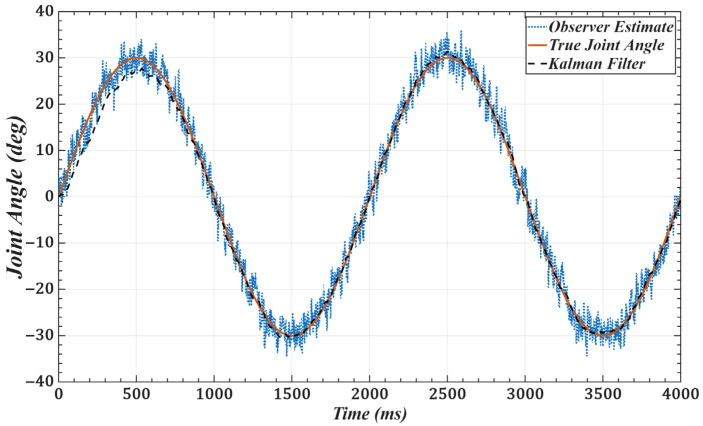
Joint 1 position with colored noise (α = 0.8): desired, noisy observer, and filtered.

**Figure 14:**
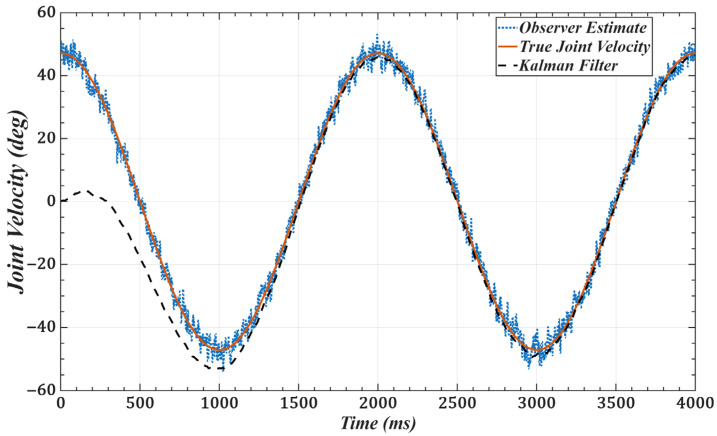
Joint 1 velocity with colored noise (α = 0.8): desired, noisy observer, and filtered.

To validate the effectiveness of the improved observer, we use joint 1 as an example and set the desired trajectory as $q_{d(t)}=\frac{\pi }{6}\sin\left(\frac{\pi }{2}t\right)$. Figure 11 and Figure 12 show the joint position and velocity after processing by the observer and filter under Gaussian white noise. Figure 13 and Figure 14 show the corresponding results under colored noise. These figures demonstrate that the designed model reference observer can effectively provide joint state information. Moreover, by incorporating the Kalman filter, the system effectively suppresses noise interference and converges to the true values after a brief delay, thereby confirming the effectiveness of the proposed method.

## 5. Experiments and Results

**Figure 15:**
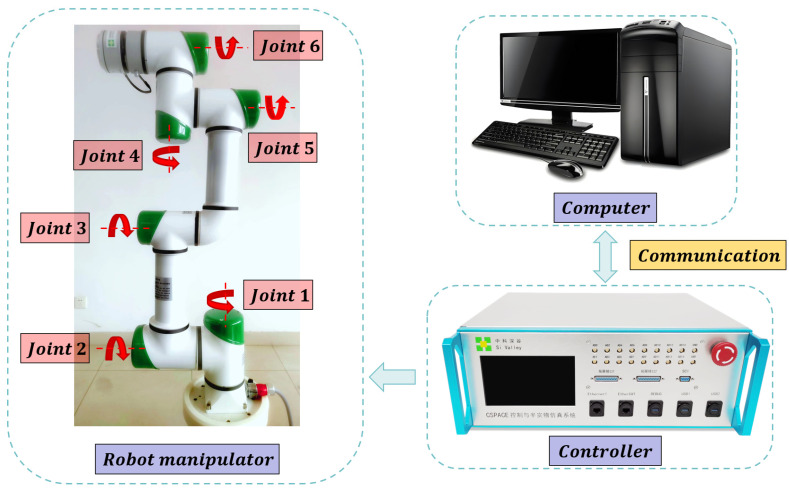
Experimental platform.

To verify the effectiveness of the proposed control system, this study employs a six-degree-of-freedom robotic manipulator developed by the Chinese company Si Valley as the research subject, whose structural and dynamic parameters can be found in [44]. The experimental platform is illustrated in Figure 15.

In terms of sensor configuration, each joint is equipped with a high-resolution absolute encoder to measure joint angle information. Regarding the hardware platform, the controller is based on a self-developed cSPACE rapid control prototyping system, with the core computing unit utilizing an ARM Cortex processor (1.2 GHz clock speed, 1 GB memory). Control algorithms are modeled and deployed in real time through the Matlab/Simulink environment. The system sampling frequency is set to 500 Hz, ensuring real-time control capability of the manipulator’s dynamic response. Communication between the joint drivers and the controller is realized via an EtherCAT bus, with a communication cycle of 0.002 s, thereby ensuring high-speed and stable data exchange.

With the above hardware and sensor configuration, the experimental platform provides reliable support for validating the proposed control algorithms and ensures the authenticity and repeatability of the experimental results.

### 5.1. Trajectory Tracking Without Load

To validate the effectiveness of the proposed control method, the experimental task trajectories were set as a circular trajectory lasting 3 s and a back-and-forth linear trajectory. Three control strategies were selected for comparison in the tracking experiments: (1) the dynamic feedforward control method [40], with feedforward torques computed using the Newton–Euler method as described in Section 3.2.; this method uses the same information and has a comparable computational load to the proposed method. (2) The adaptive time-delay control method [42], which achieves adaptive control through online adjustment of the control gain $\bar{M}$, with a relatively higher computational complexity. (3) The proposed robust time-delay control method. The first two methods are relatively recent control strategies and have comparable information usage and computational complexity to the proposed approach, making them reasonable baselines for a fair comparison.

**Figure 16:**
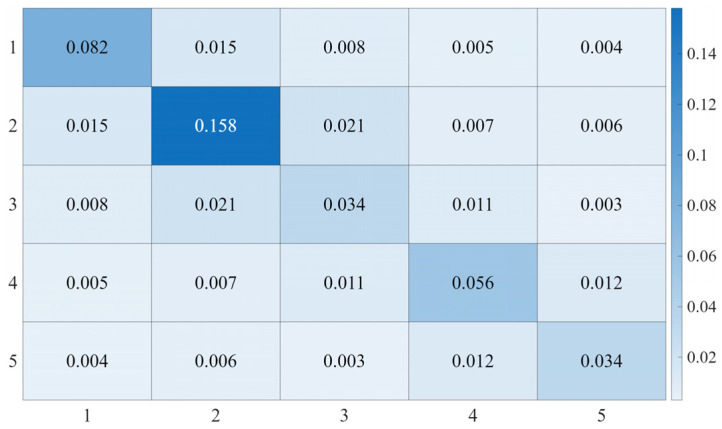
Heatmap of the Control Gain Matrix $\bar{M}$ for Circular Trajectory.

Since the *6*-*th* joint does not affect the end-effector trajectory, the analysis in this study focuses only on the first five joints. For the circular trajectory, the controller parameters are set as follows: $K_{p}=diag(400,600,300,150,150)$, $K_{v}=diag(38,45,30,15,15)$, and the control gain matrix $\bar{M}$ is shown in Figure 16.

**Figure 17:**
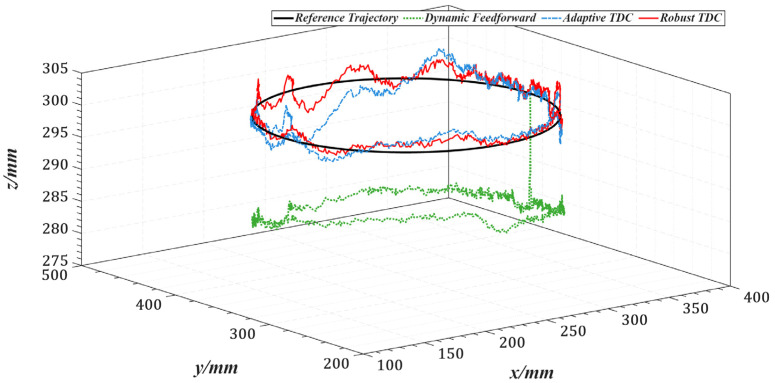
End-effector trajectories in Cartesian space under three control strategies.

**Figure 18:**
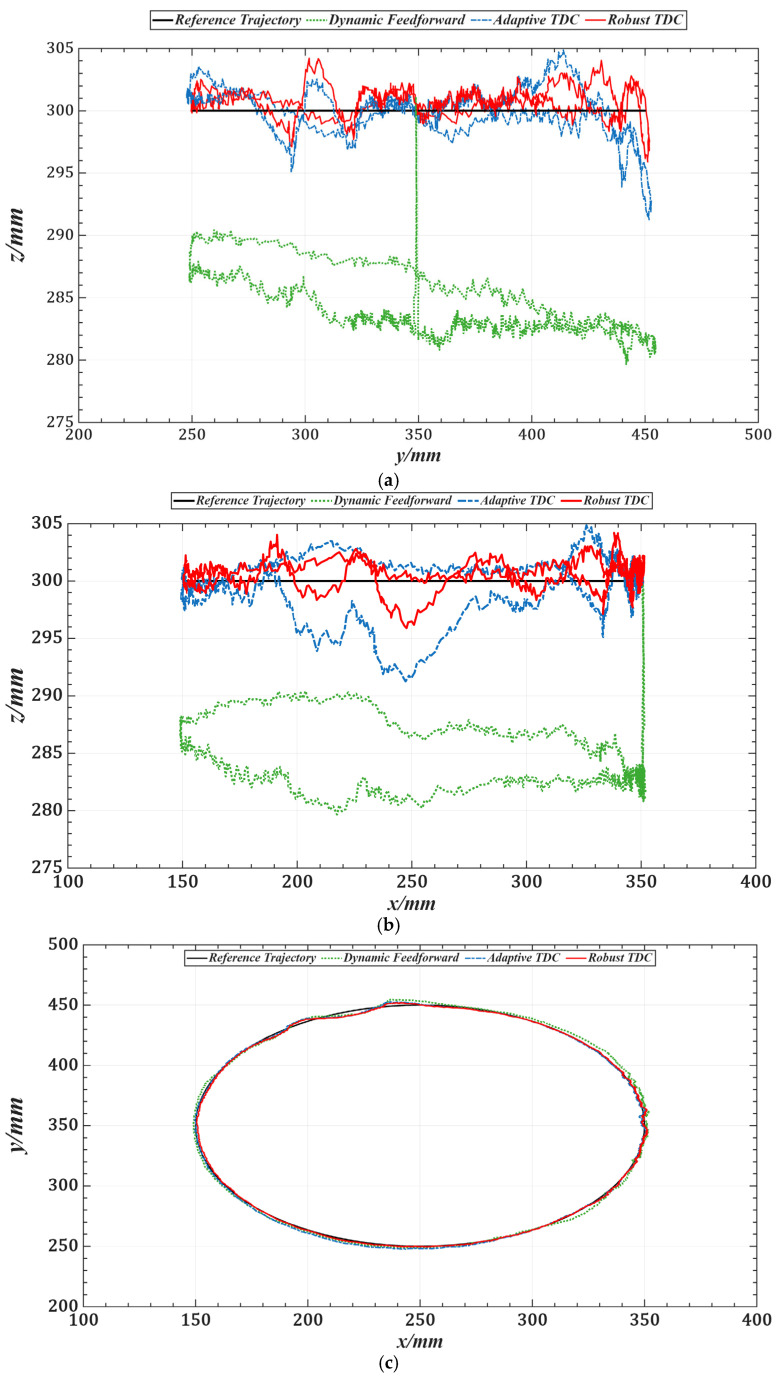
X, Y, and Z directional views of end-effector trajectories under three control strategies. (**a**) X-direction view. (**b**) Y-direction view. (**c**) Z-direction view.

Figure 17 illustrates the overall tracking performance of the end-effector along a circular trajectory in the Cartesian coordinate system for the three control methods. Figure 18 further presents the end-effector tracking from the X, Y, and Z directions to provide a more intuitive visualization.

**Figure 19:**
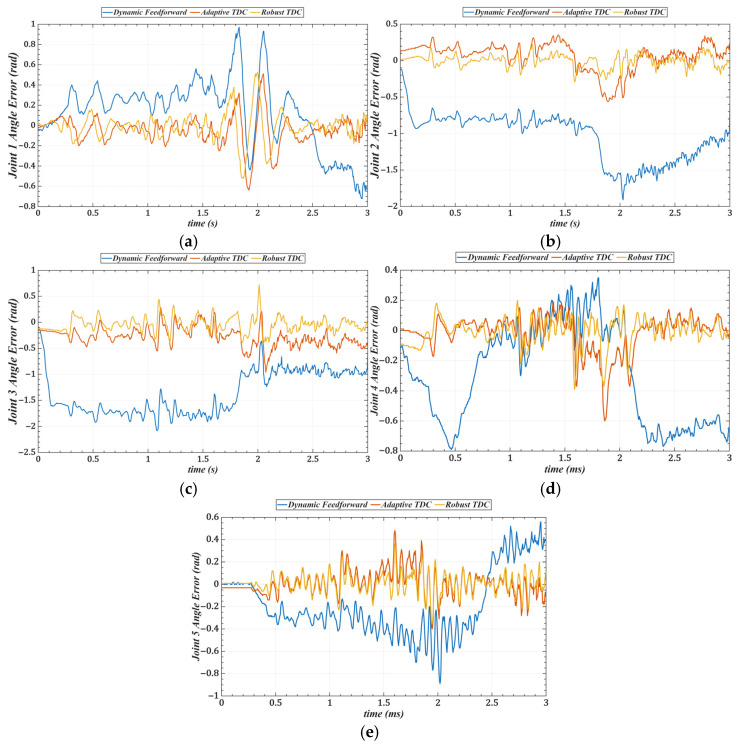
Tracking errors of joints 1–3 in joint space under three control strategies. (**a**) Joint 1. (**b**) Joint 2. (**c**) Joint 3. (**d**) Joint 4. (**e**) Joint 5.

Table 3 lists the root mean square errors (RMSE) of the end-effector tracking along the circular trajectory in the Cartesian space for the three methods. Figure 19 shows the tracking errors of joints 1 to 5 in the joint space, and Table 4 summarizes the corresponding RMSE values for these joints.

**Figure 20:**
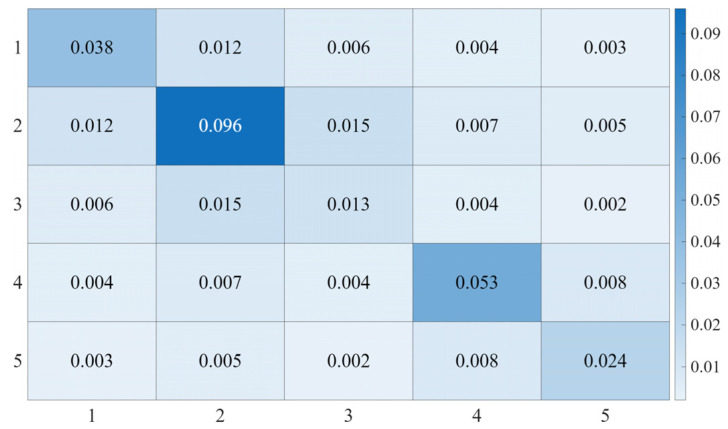
Heatmap of the Control Gain Matrix $\bar{M}$ for the back-and-forth linear trajectory.

**Figure 21:**
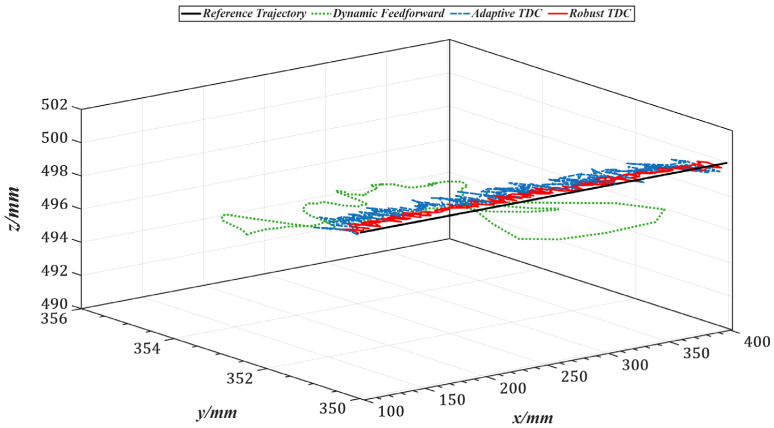
End-effector trajectories in Cartesian space under three control strategies.

**Figure 22:**
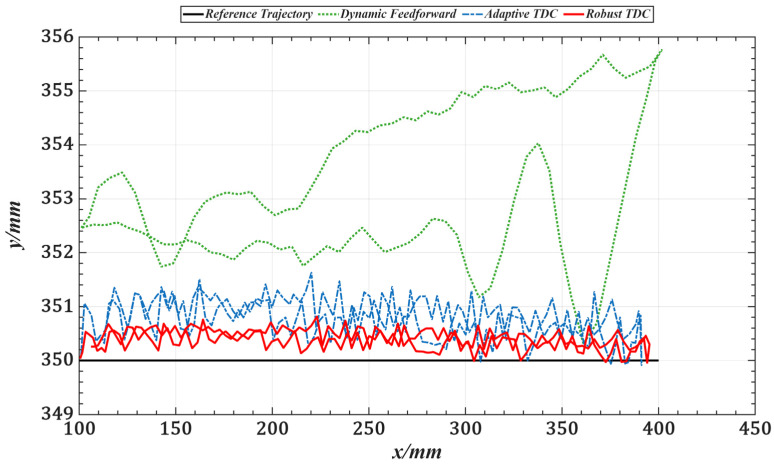
Z directional views of end-effector trajectories under three control strategies.

**Figure 23:**
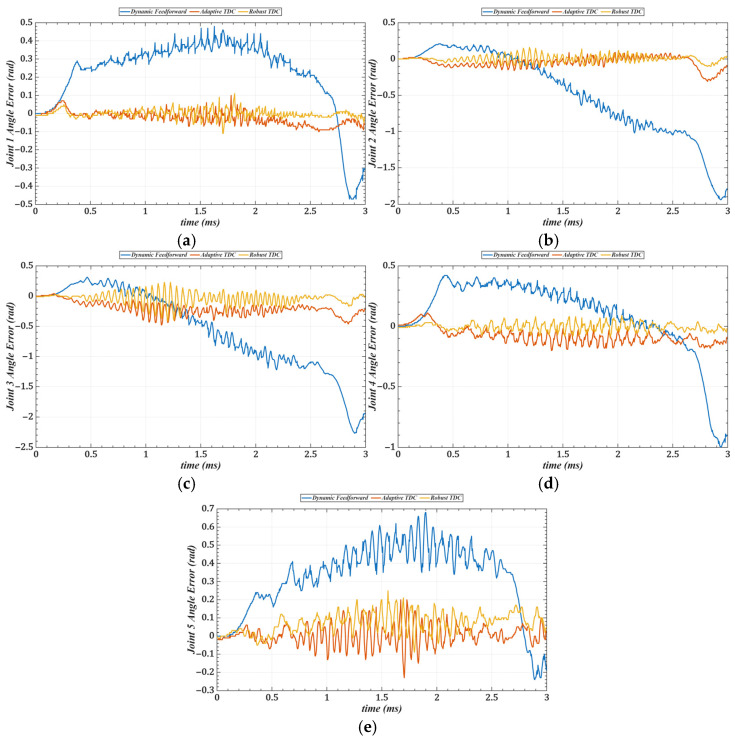
Tracking errors of joints 1–3 in joint space under three control strategies. (**a**) Joint 1. (**b**) Joint 2. (**c**) Joint 3. (**d**) Joint 4. (**e**) Joint 5.

For the back-and-forth linear trajectory, the controller parameters are set as follows: $K_{p}=diag(400,600,300,150,150)$, $K_{v}=diag(38,45,30,15,15)$, and the control gain matrix $\bar{M}$ is shown in Figure 20. Figure 21 illustrates the overall tracking performance of the end-effector along the back-and-forth linear trajectory in the Cartesian coordinate system for the three control methods. Figure 22 further presents the end-effector tracking from the Z directions, providing a more intuitive visualization of the tracking performance. Figure 23 shows the tracking errors of joints 1 to 5 in the joint space, and Table 5 and Table 6 summarizes the corresponding RMSE values for these joints under the three control methods.

### 5.2. Trajectory Tracking with Load

**Figure 24:**
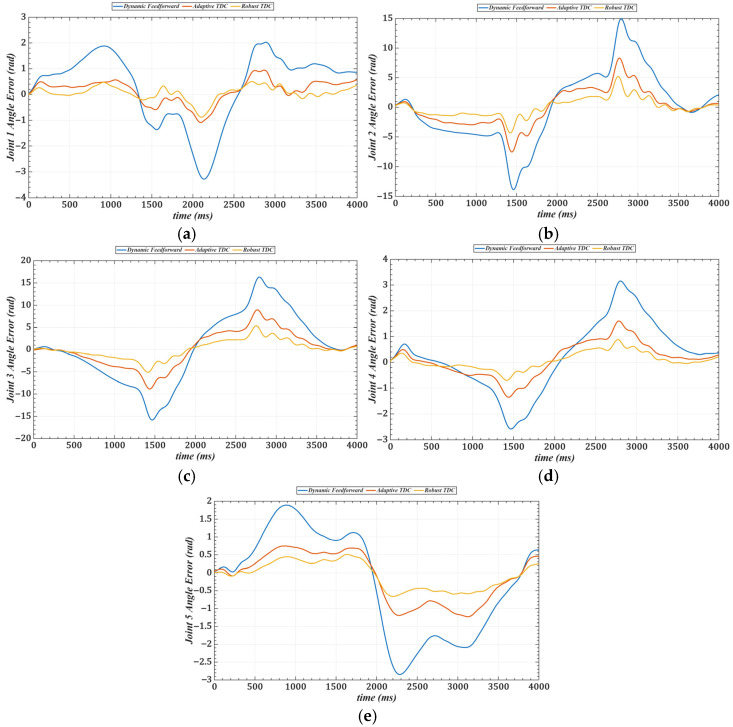
Tracking errors of joints 1–3 in joint space under three control strategies with load. (**a**) Joint 1. (**b**) Joint 2. (**c**) Joint 3. (**d**) Joint 4. (**e**) Joint 5.

To validate the trajectory tracking performance of the proposed method under loaded conditions, a 2 kg metal block is selected as the end-load and mounted at the end of the manipulator. In the experiment, the first five joints simultaneously track the desired trajectory: $q_{d(t)}=\frac{\pi }{6}\sin\left(\frac{\pi }{4}t\right)-\frac{\pi }{12}\sin\left(\frac{\pi }{6}t\right)$. Three control strategies are employed for trajectory tracking. Figure 24 shows the tracking performance in joint space under the three control strategies with the load applied. Table 7 provides the RMS tracking errors for each method.

## 6. Discussion

High-precision and structurally simple control methods for robotic manipulators can significantly enhance the efficiency of industrial production. This paper proposes a robust time-delay control strategy based on an improved state observer, which not only features low computational complexity and ease of implementation but also demonstrates significant advantages in trajectory tracking performance.

One of the key advantages of the proposed method is its low computational complexity. The online computation is equivalent to that of a PID controller, while the more complex calculations, such as gain optimization, are pre-computed offline. This eliminates the need for tedious real-time adjustments, leading to a more efficient system. As shown in Figure 6 and Figure 7 and Table 1 and Table 2, compared with scaled gains, the control gains determined by the proposed method substantially improve trajectory tracking accuracy, achieving superior control performance. Additionally, the method effectively addresses the unmeasurable state problem while exhibiting strong noise robustness. As illustrated in Figure 11, Figure 12, Figure 13 and Figure 14, the proposed approach provides high-quality state information.

Compared with the dynamics feedforward control strategy, Table 3 and Table 4 show that the proposed method reduces the RMS errors in Cartesian and joint space by an average of approximately 87%, indicating that it can more fully exploit the system’s dynamic information. Furthermore, the introduced time-delay control law achieves better closed-loop performance than conventional feedback control strategies. Compared with the adaptive time-delay control strategy, the proposed method reduces RMS errors by an average of about 45% under the same conditions, demonstrating that the control gains obtained through offline optimization can replace the real-time adaptive adjustment process, thereby reducing computational delay and improving response speed.

Furthermore, to verify the practicality of the proposed method, experiments under a loaded condition were conducted. The load, a 2 kg metal block, was placed at the end of the manipulator. The results from these experiments confirm that the proposed method performs effectively under varying loading conditions, maintaining accuracy and robustness.

In summary, the experimental results validate the effectiveness of the proposed robust time-delay control strategy for joint trajectory tracking and indicate that the method possesses high practicality and reliability for achieving high-precision control of robotic manipulators.

## 7. Conclusions

High-precision control of robotic manipulators is of great significance in industrial production. However, achieving efficient and reliable trajectory tracking still faces challenges such as tedious tuning of control gains and unmeasurable system states. This paper proposes a robust time-delay control strategy based on an improved state observer. By combining offline optimization of control gains with a model reference observer–Kalman filter fusion scheme, the effectiveness of the proposed method is validated through simulations and experiments on a six-axis robotic manipulator. Experimental results demonstrate that the method achieves significant improvements in both trajectory tracking accuracy and state estimation quality.

The main contributions of this work are as follows:

1. **Efficient control gain design:** The proposed time-delay control gain determination method can be completed offline, without relying on engineer experience or introducing additional tuning parameters, enabling rapid determination of optimal control gains.
2. **Accurate state estimation:** By integrating a model reference observer with a Kalman filter, measurement noise is effectively suppressed, allowing high-precision estimation of joint states.
3. **Strong engineering applicability:** The method features a simple structure and low computational cost, making it suitable for practical implementation in robotic manipulator systems and offering substantial potential for wider application.

Future work will focus on extending the method to multi-robot cooperative control and further optimizing the design of the state observer to improve state estimation accuracy under high-speed dynamic tasks. In addition, the robustness of the control strategy will be evaluated in more complex or uncertain environments.

## Figures and Tables

**Table 1:** Tracking errors of Joint 1 under different control gains.

Control Gain	${0.8\bar{M}}_{i}$	${1.2\bar{M}}_{i}$	${\bar{M}}_{i}$
Mean Error	3.89 × 10^−2^	1.07 × 10^−2^	2.35 × 10^−3^
RMS Error	4.89 × 10^−3^	3.61 × 10^−3^	2.70 × 10^−4^

**Table 2:** Tracking errors of Joint 2 under different control gains.

Control Gain	${0.8\bar{M}}_{i}$	${1.2\bar{M}}_{i}$	${\bar{M}}_{i}$
Mean Error	2.73 × 10^−2^	1.19 × 10^−2^	2.26 × 10^−3^
RMS Error	7.70 × 10^−3^	2.86 × 10^−3^	4.17 × 10^−4^

**Table 3:** RMS errors of end-effector tracking in Cartesian space.

RMS/mm	Dynamic Feedforward	Adaptive TDC	Robust TDC
X-direction	1.5747	0.3245	0.2430
Y-direction	2.7832	0.5947	0.4356
Z-direction	6.1254	1.1361	0.8711
Composite RMS	6.9024	1.3485	0.9564

**Table 4:** RMS errors of joints 1–5 in joint space.

RMS/rad	Dynamic Feedforward	Adaptive TDC	Robust TDC
Joint 1	0.2775	0.1189	0.1062
Joint 2	0.8428	0.1554	0.0671
Joint 3	1.0997	0.2551	0.1175
Joint 4	0.5303	0.1211	0.0799
Joint 5	0.3398	0.1447	0.1094

**Table 5:** RMS errors of end-effector tracking in Cartesian space.

RMS/mm	Dynamic Feedforward	Adaptive TDC	Robust TDC
X-direction	1.7453	0.9738	0.5684
Y-direction	3.1874	0.7898	0.3949
Z-direction	3.4349	0.4432	0.2216
Composite RMS	5.0004	1.3298	0.7267

**Table 6:** RMS errors of joints 1–5 in joint space.

RMS/rad	Dynamic Feedforward	Adaptive TDC	Robust TDC
Joint 1	0.3250	0.0616	0.0257
Joint 2	0.9343	0.6524	0.0451
Joint 3	1.0676	0.2056	0.0927
Joint 4	0.5229	0.0870	0.0392
Joint 5	0.3478	0.1010	0.0702

**Table 7:** RMS errors of joints 1–5 in joint space.

RMS/rad	Dynamic Feedforward	Adaptive TDC	Robust TDC
Joint 1	0.1538	0.0829	0.0742
Joint 2	0.4345	0.2596	0.1923
Joint 3	0.8078	0.4397	0.2738
Joint 4	0.3568	0.1924	0.1280
Joint 5	0.3840	0.2065	0.1375

## Data Availability

The data that support the study are available from the corresponding author, J.D., upon reasonable request. The data are not publicly available due to privacy.
